# Effects of collagen membrane on bone level and periodontal status of adjacent tooth after third molar surgery: a randomized controlled trial

**DOI:** 10.1186/s13005-023-00351-8

**Published:** 2023-03-25

**Authors:** Adnan Kilinc, Mert Ataol

**Affiliations:** 1 Private Kılınç Dental Clinic, 25040, Erzurum, Turkey; 2 Private Zoom Dental Clinic, 06530 Ankara, Turkey

**Keywords:** Bone Level, Periodontal Status, Collagen Membrane, Healing Type, Partially Impacted Third Molar

## Abstract

**Background:**

The periodontal status and distal bone level of the adjacent second molar can be negatively affected by the surgical extraction of an impacted lower third molar. Absorbable materials have some benefits, including enhancing primary wound coverage and promoting wound healing through isolation, clotting, wound stabilization and haemostasis. This study set out to compare primary and secondary healing and collagen-membrane-based primary healing after surgical removal of partially erupted impacted third molars (3Ms), evaluating the distal alveolar bone level (ABL) and periodontal status of the adjacent second molars (2Ms).

**Methods:**

Patients who met the inclusion criteria were randomized into three groups: secondary healing (*n* = 28), primary healing (*n* = 27) and membrane-based primary healing (*n* = 29). Digital panoramic radiographs were obtained preoperatively (T1) and three months postoperatively (T2). The distances between the cemento-enamel junctions and the alveolar bone crests on the distal aspects of the adjacent 2Ms were measured using calibrated radiograph measurement software. The pocket depth and plaque index measurements were performed preoperatively and three months postoperatively. The periodontal plaque index (PPI) scores were registered on the distal aspects of the 2Ms, and the mean values were used.

**Results:**

Three of the applied healing types positively affected periodontal pocket depth (PPD) and periodontal index values (*p* < 0.05). In terms of the ABL of the adjacent 2Ms, primary healing (*p* < 0.05) and membrane-based primary healing (*p* < 0.05) had superior results to secondary healing.

**Conclusion:**

Membrane use is promising for the distal bone gain and periodontal status of the adjacent 2M.

**Trial registration:**

This clinical study was registered by the Australian New Zealand Clinical Trials Registry, with the trial number ACTRN12618001551280.

## Background

A serious condition that can arise after the surgical extraction of an impacted lower third molar (3M) is the deterioration of the periodontal status of the adjacent second molar (2M). This condition can take a chronic course that affects the long-term stability and survival of the 2M. A controversial but predominant opinion is that bone loss may occur on the 2M’s distal aspect. In such cases, the 2M’s cementum can be exposed, and the condition may even require tooth extraction [1–3].

Many researchers have focused on factors such as flap design [4–6], sutures [7], healing type [8] and adjunctive periodontal regenerative therapy [3, 9, 10] for minimizing this complication. However, it remains unclear whether primary or secondary intention healing can influence the distal bone level and periodontal status of the adjacent 2Ms. In our literature review, the studies concerning healing type and bone level were not radiological, but were clinical studies in which measurements were taken using periodontal probes or the periodontal index [3, 7, 8, 11, 12]. Therefore, there was a need for a study evaluating distal bone level with radiological and clinical measurements.

Healing type has been suggested to periodontal status of the 2Ms. With regards to partially erupted impacted molars, primary intention healing is only possible with a sliding flap, which would be difficult compared to fully impacted teeth without tissue loss. In these situations, primary wound healing can be supported with various materials [8].

Absorbable materials have some benefits, including enhancing primary wound coverage and promoting wound healing through isolation, clot and wound stabilization and haemostasis [13]. Absorbable collagen membranes have been used in both the medical and dental fields for decades. To our knowledge, no previously published study has evaluated the long-term effects of collagen membranes on distal bone regeneration and the periodontal tissues of adjacent 2Ms after the surgical removal of partially erupted impacted 3Ms.

This study intended to evaluate the following hypothesis: Is the administration of collagen membrane after surgical removal of partially erupted impacted 3Ms effective on periodontal healing? This study compared primary and secondary healing and collagen-membrane-based primary healing after surgical removal of partially erupted impacted 3Ms, evaluating the distal alveolar bone level (ABL) and periodontal status of the adjacent 2Ms. Short-term results of this study were presented in a previous manuscript.

## Methods

This prospective, randomized controlled study included patients with vertical or mesioangular partially impacted 3Ms. Patients were excluded if they had medical illnesses or took medication that could influence the course of postoperative wound healing. The clinical study was registered by the Australian New Zealand Clinical Trials Registry, with the trial number ACTRN12618001551280. Patients between the ages of 18 and 30 were included. Patients were excluded from randomization if they had acute pericoronitis, a pre-existing abscess or cellulites, any restorations or carious lesions on the distal surfaces of the 2Ms, or pathological conditions associated with the 3Ms.Oral contraceptive users and smokers were also excluded.

This study’s parameters included gender, duration of surgery, surgical difficulty and the position of the 3Ms. Surgical difficulty was recorded immediately after the procedure and evaluated using a four-class difficulty scale, as follows: 1) extraction requiring forceps only; 2) extraction requiring osteotomy; 3) extraction requiring osteotomy and coronal sectioning; and 4) complex extraction(root sectioning) [14]. The level of impaction of the 3Ms was classified as being either on the level of the occlusal plane or between the occlusal plane and the cervical line of the 2 M (below the occlusal plane), according to Pell and Gregory’s classification. 3 M angulations were classified on a two-class scale according to Winter’s classification, as either 1 (mesioangular) or 2 (vertical). Digital panoramic radiographs were obtained to ensure the similarity of the impaction types according to angulations and relationship to the occlusal plane [15].

In this study methodology, the groups were selected randomly and results were evaluated by another researcher blinded to the groups. Patients were randomized to three groups by the opaque envelope method for blind selection. With this method, a total of 30 Group cards, 10 for each group, was prepared and sealed in opaque envelopes. After each impacted tooth was extracted, one envelope was randomly selected to determine the patient’s operative group. The tissue was closed and sutured according to the patient’s group. These groups were the secondary closure (SC) group, distinguished by partial closure of the extraction site to provide secondary healing; the primary closure (PC) group, distinguished by sliding the flap and suturing primarily to total closure of the extraction site; and the membrane-based primary closure (MBPC) group, distinguished by placing a collagen membrane and sliding the flap to total closure of the extraction site.

Surgical operations were carried out by a single surgeon with each patient under local anesthesia, which was achieved with up to4 mL of Articaine-HCl and a 1:100,000 ratio of epinephrine (Ultracaine D-S Forte, Aventis). An incision was made from the anterior border of the mandibular ramus, extending to the distal surface and the buccal gingivodental sulcus of the 2 M. The incision was continuous, with vertical incision oblique into the mandibular vestibular fornix, aligned with the2M’s mesiobuccal cusp. A full-thickness mucoperiosteal triangular flap was elevated. If necessary, osteotomy was performed under constant irrigation.

The wound closure was performed with atraumatic silk sutures, depending on the group. In the SC group, the flap was positioned to its former position by a single suture distal to the 2 M, leaving a gap. If necessary, a wedge of mucosa was removed. In the PC group, the sliding flap was sutured. In the MBPC group, the extraction socket was closed with a resorbable collagen membrane (Evolution, Osteobiol-Tecnoss, Italy), and the sliding flap was sutured primarily.

The patients were given standard postoperative medication; antibiotics (amoxicillin + clavulanic acid) for five days, nonsteroidal anti-inflammatory drugs (dexketoprofen trometamol) for 3 days and mouthwash (with 0.12% chlorhexidine) for two weeks, according the body mass of each patient. Additionally, an oral health education intervention was presented. The sutures were removed after seven days.

The postoperative measurements and the study’s outcomes were examined and determined by another researcher blinded to the group of patients. One of the outcome variables was the periodontal status of the 2Ms. Periodontal measurements were performed preoperatively (T1) and three months postoperatively (T2). The pocket depths and plaque index scores of the2M’s distobuccal (DB), distolingual (DL), midbuccal (MidB) and midlingual (MidL) surfaces were recorded. All periodontal pocket depth (PPD) measurements were performed in millimetres, using William’s periodontal probe (Aesculap AG&Co., Tuttlingen, Germany).To assess the periodontal plaque index (PPI) scores, the Silness&Löe plaque index was registered on the distal aspects of the 2Ms,and the mean values were used.

To evaluate the difference in bone level, which was the other outcome variable, digital panoramic radiographs in standardized position were obtained preoperatively and three months postoperatively by a single panoramic x-ray device. Cemento-enamel junctions (CEJs) were used as reference points, and the distances between the CEJ and the alveolar bone crest on the distal aspect of the adjacent 2M were measured in millimeters using calibrated radiograph measurement software (MedData Medical Software, Ankara, Turkey). All radiological measurements were performed twice and independently by another two clinicians who were not involved in this study, and mean values were recorded preoperatively (T1) and three months postoperatively (T2). Among this study’s primary outcomes were the differences (positive or negative) between the pre-and postoperative radiographic measurements.

### Statistical analysis

Sample size was specified by using a power analysis. To detect a significant difference at an effect size of 0.69 and power level of 80% with a 95% confidence level, at least 30 patients were required for each group.

Statistical analysis was performed using the SPSS statistical software package, version 20.0 (IBM, Chicago, IL, USA). The normalities of the distributions were tested using the Kolmogorov–Smirnov test. The Wilcoxon signed rank test was used for intragroup comparisons. For intergroup comparisons, the Kruskal–Wallis or chi-square test was used, and for post hoc tests, the Bonferroni correction was used.

## Results

Ninety patients were included in this study, but four of them quit the study and two were excluded because of early post-operative complications (wound opening). Eighty-four patients completed the study. The sample included 23 men (27.4%) and 61 women (72.6%), resulting in a male-to-female ratio of 1:2.65. Table 1compares the three groups based on factors that could have affected the outcome variables. These results show no statistically significant differences (*p* > 0.05) except in operation time (*p* = 0.002) (Table 1).

**Table 1:** Study Variables and Descriptive Statistics

	**SC**	**PC**	**MBPC**	
	**Mean or n**	**Mean or n**	**Mean or n**	**(p)**
**Age (Std)**	22.18	24.03	23.79	0.196
	(4.29)	(4.49)	(5.48)	
**Gender**				
Female	21	20	20	0.859
Male	7	7	9	
Total	28	27	29	
**Angulation**				
Vertical	20	16	22	0.384
Mesioangular	8	11	7	
**Depth**				
Occlusal plane	18	15	17	0.799
Below occlusal plane	10	12	12	
**Surgical Difficulty**				
1	15	13	14	0.614
2	5	5	7	
3	6	5	2	
4	2	4	6	
**Operation Time**	10.89	15.15	15.86	0.002^*^

*Std* Standard Deviation; ^*^p < 0.05

The intragroup statistics, as well as the plaque index scores and probing depths taken preoperatively and three months postoperatively, are listed in Table 2. These results show that DB and DL pocket depths were statistically significantly reduced for all three groups (*p* < 0.05). MidB and MidL pocket depths were statistically significantly reduced in the MBPC group (*p* = 0.000 and *p* = 0.012, respectively), but not in the SC or PC group (*p* > 0.05). Plaque index and oral health positively differed for all three groups (*p* = 0.000). There were no statistically significant differences among the groups according to intergroup comparisons of PPD and PPI (*p* > 0.05).Furthermore, mobility of the adjacent second molar was not observed in any of the patients followed in the study.

**Table 2:** Means, standard deviations and comparisons of periodontal measurements

	**SC**			**PC**			**MBPC**			
	T1	T2	p	T1	T2	p	T1	T2	p	***p***
	Mean (Std)	Mean (Std)		Mean (Std)	Mean (Std)		Mean (Std)	Mean (Std)		
**DB**	3.57 (1.79)	2.29 (0.67)	0.001*	4.11 (1.78)	2.54 (0.75)	0.000*	3.86 (1.46)	2.26 (0.81)	0.000*	*0.531*
**DL**	3.50 (1.75)	2.16 (0.64)	0.000*	4.19 (1.86)	2.41 (1.01)	0.000*	3.31 (1.51)	1.90 (0.47)	0.000*	*0.429*
**MidB**	1.79 (0.63)	1.48 (0.50)	0.056	1.89 (0.64)	1.61 (0.74)	0.070	1.93 (0.65)	1.40 (0.49)	0.000*	*0.500*
**MidL**	1.86 (0.52)	1.66 (0.55)	0.065	2.04 (0.94)	1.57 (0.74)	0.081	1.76 (0.69)	1.40 (0.49)	0.012*	*0.295*
**PPI**	1.39 (0.88)	0.61 (0. 69)	0.000*	1.52 (0.85)	0.56 (0.64)	0.000*	1.34 (0.77)	0.55 (0.69)	0.000*	*0.608*

*Std* Standard Deviation, ^*^p < 0.05, *T1* Preoperatively, *T2* Three Months Postoperatively, *DB* Distobuccal Pocket Depth, *DL* Distolingual Pocket Depth, *MidB* Midbuccal Pocket Depth, *MidL* Midlingual Pocket Depth, *PPI* Periodontal Plaque Index

**Fig. 1:**
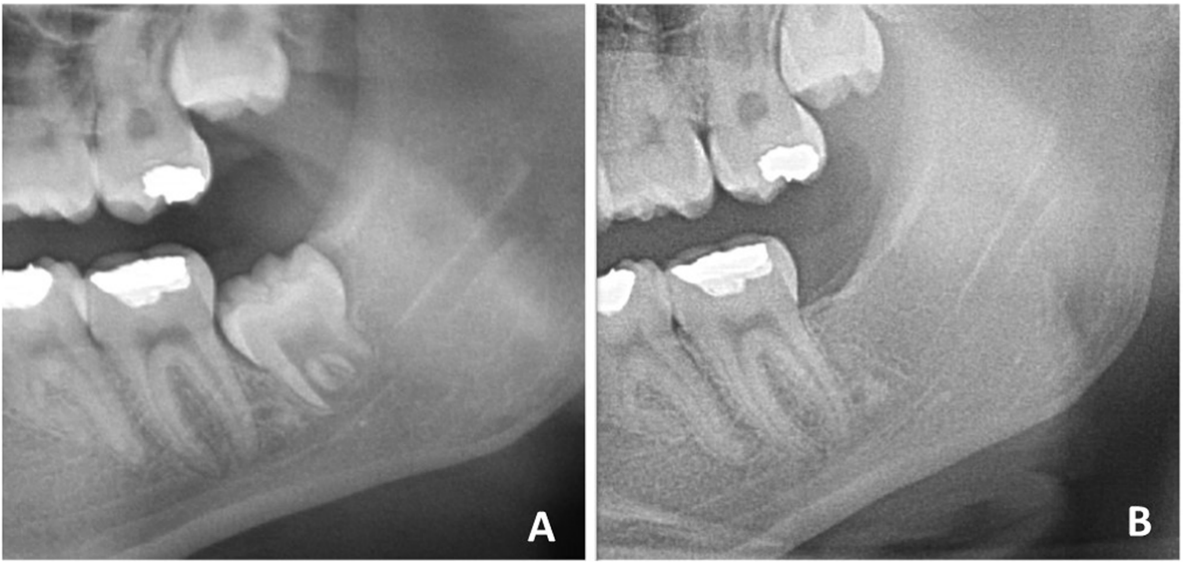
Radiographic images of a MBPC group patient. **A** Preoperative; **B **Three Months Postoperative

Table 3 shows the height differences in the ABL for all groups, as measured preoperatively and three months postoperatively in the radiographs at the 2Ms’distal surfaces. These results show that there was no statistically significant difference in distal bone level in the SC group(*p* = 0.08).On the other hand, there were statistically significant differences in the PC group(*p* = 0.01) and MBPC group(*p* = 0.000) (Fig. 1). In the intergroup comparison, there was a statistically significant difference among the three groups (*p* = 0.000).In binary comparisons, although the change in distal bone level scores was statistically significantly superior in the MBPC and PC groups to that in the SC group, there was no statistically significant difference between the PC and MBPC groups (*p* = 0.053).

**Table 3:** Means, standard deviations and comparisons of alveolar bone level

	**SC**	**PC**	**MBPC**
	Mean (Std)	Mean (Std)	Mean (Std)
T1	2.36 (1.31)	3.13 (1.95)	2.86 (1.41)
T2	2.73 (1.55)	2.41 (1.03)	1.50 (0.44)
**p**	0.088	0.01*	0.000*
T2-T1	− 0.38 (1.11)	0.72 (1.30)	1.36 (1.24)
**Intergroup Comparison**	**Binary Comparisons**		
	**SC/PC**	**SC/MBPC**	**PC/MBPC**
p	p	p	p
0.000*	0.001*	0.000*	0.053

*Std* Standard Deviation; ^*^p < 0.05; *T1* Preoperatively, *T2* Three Months Postoperatively

## Discussion

Various factors can influence the surgical difficulty and post-operative complications of an impacted lower third molar, such as position, width, bone and soft tissue coverage, associated pathologies, number of roots, root angulation-dilaceration, gender, age, body mass index, surgeon’s experience, contact with adjacent second molar, etc. [16–18] This study’s limitation is that its results are compared in only terms of closure method when, in fact, they depend on many variables. Because there are so many variables, it is very important to determine strict inclusion and exclusion criteria in order to avoid bias, and to compare accurate groups and produce reliable results when conducting a randomized controlled study.

The healing result of a single site is dependent on a number of factors at baseline that may vary from site to site or person to person [19].For this reason, taking into account the randomized nature of the study, the inclusion criteria were rigorously planned and applied to make the groups comparable. There was no statistically significant difference among the groups in terms of age, gender, angulation, depth or surgical difficulty (*p* > 0.05).The results showed that all three groups were comparable in terms of periodontal healing and bone level.

Postoperative complications as pain, swelling, limitation in mouth opening, bleeding, dehiscence, and dry socket were evaluated in a previous manuscript. The results showed that there was no statistically significant difference associated with collagene membrane usage when compared with the primary closure group [20]. Some other short term complications have been reported in the literature such as; nerve damage, fracture, tooth displacement, damage on adjacent second molar teeth, etc. [21]. In a systematic review that considers short-term results, it is suggested that the secondary closure has a favorable effect on pain, facial swelling, and trismus after the surgical removal of impacted third molars [21]. Literatures on long-term complications often focus on the periodontal status of the adjacent second molar [3, 22].

In studies comparing primary and secondary wound healing and investigating the effect of extracting partially impacted 3Ms on the periodontal health of 2Ms,deep periodontal pockets have been reported [2]. By contrast, other studies have reported a decrease in the PPD at the distal aspect of the 2Ms. [6, 11] Referring to these results, the investigators reported that the periodontal problems in the soft tissues around the 2Ms after 3 M surgery were not related to the flap technique, and that this technique did not affect the 2Ms’ periodontal health [22]. Also, PPD value has a linear relationship with the number of anaerobic organisms in the mouth and is associated with certain factors such as oral hygiene. [23, 24] For this reason, PPD values may not always correspond well with ABL values [24].

Hashemi et al. [7] and Korkmaz et al. [8] showed that at their three-month follow-up, neither the primary nor secondary closure group showed a statistically significant difference between the preoperative and postoperative measurements of PPD around the 2Ms. In addition, the authors reported that PPD values were less than 3 mm in both groups. Cortell-Ballester et al. [9] evaluated resorbable collagen membranes during fully impacted mandibular 3 M surgery and reported that this reduced the distal PPD of the adjacent 2Ms. In the present study, statistically significant decreases in the DB, DLPPD and PPI were observed at the three-month follow-up with all three healing approaches. However, there were no statistically significant differences among the groups in PPD and PLI (*p* < 0.05).

On the other hand, the scientific data about dimensional bone changes occurring after 3 M surgery distal to the adjacent 2Mswas limited in the studies investigating primary and secondary wound healing.

In line with our findings, Aimetti et al. [25] evaluated the effects of the placement of a membrane distal to the adjacent 2Ms after 3 M surgery and reported statistically significant bone gain. Sammartino et al. [12] showed successful results on bone level with the usage of a collagen membrane with platelet-rich plasma. Cortell-Ballester et al. [9] conducted a study using absorbable collagen membrane and showed that its use supported healing and increased bone formation at a three-month follow-up. In the present study, there was a statistically significant difference between the PC and SC groups and between the SC and MBPC groups (*p* < 0.05).These results suggest that primary healing methods may be advantageous in terms of ABL. On the other hand, there was a statistically non-significant positive result on bone level between the primary healing and membrane-based primary healing groups (*p* = 0.053), and these three-month follow-up results are seen as promising for bone gain on the distal aspects of adjacent 2Ms.

## Conclusion

According to this study, all three healing types applied in this study positively affected PPD and PPI values. This result may be related to the easier removal of plaque at the 2Ms’ distal aspects after extraction. On the other hand, in terms of the 2Ms’ ABLs, primary healing and membrane-based primary healing had superior results to secondary healing. In our opinion, especially when the patient’s bone level is insufficient, primary healing is advantageous and preferable. Additionally, the use of resorbable collagen membranes is a promising approach after the surgical extraction of impacted lower 3Ms, as it promotes bone regeneration and improves the periodontal status of the adjacent 2Ms.

## Data Availability

This clinical study was registered by and the data of this study could be reached at Australian New Zealand Clinical Trials Registry with the trial number of ACTRN12618001551280.

## Abbreviations

3M: Third molar
2M: Second molar
ABL: Alveolar bone level
CEJ: Cemento-enamel junction
DB: Distobuccal
DL: Distolingual
MBPC: Membrane-based primary closure
MidB: Midbuccal
MidL: Midlingual
PC: Primary closure
PPD: Periodontal pocket depth
PPI: Periodontal plaque index
SC: Secondary closure
T1: Preoperative time
T2: Postoperative time
